# Subsistence strategy changes during the Middle to Upper Paleolithic transition reveals specific adaptations of Human Populations to their environment

**DOI:** 10.1038/s41598-019-50647-6

**Published:** 2019-11-01

**Authors:** William Rendu, Sylvain Renou, Marie-Cécile Soulier, Solange Rigaud, Morgan Roussel, Marie Soressi

**Affiliations:** 1 PACEA, UMR 5199, CNRS, Université de Bordeaux, Ministère de la Culture et de la Communication (MCC), F-33400 Pessac, France; 2 Department of Anthropology, New York University, CSHO, New York, NY 10003 USA; 3 TRACES, UMR 5608, CNRS, Université Toulouse Le Mirail, F-31058 Toulouse Cedex 9, France; 4 Faculty of Archaeology, Leiden University, 2333CC Leiden, Netherlands; 5 Department of Human Evolution, Max Planck Institute for Evolutionary Anthropology, Deutscher Platz 6, 04103 Leipzig, Germany

**Keywords:** Archaeology, Archaeology, Palaeoecology

## Abstract

The transition from Middle to Upper Paleolithic is a major biological and cultural threshold in the construction of our common humanity. Technological and behavioral changes happened simultaneously to a major climatic cooling, which reached its acme with the Heinrich 4 event, forcing the human populations to develop new strategies for the exploitation of their environment. The recent fieldwork at Les Cottés (France) transitional site offers a good opportunity to document subsistence strategies for this period and to provide for the first time high-resolution insights on its evolution. We present the results of the complete zooarchaeological and taphonomic analysis of the transitional sequence, associated with a large regional synthesis of the subsistence strategy evolution during the Middle to Upper Paleolithic. We conclude that, while there is no major change in the hunting strategies, the butchery activities evolved in strict correlation with the development of range weapons. In addition, the demise of carnivore seems to be a consequence of the human pressure on the environment. Our study demonstrates how the faunal component of the environment became a structuring element of the human social organization, being at the base of future cultural evolutions.

## Introduction

The Middle to Upper Paleolithic transition is well known for the major demographic shift that occurred with the arrival of anatomically modern humans (AMH) in Europe, their interbreeding with the local Neanderthal populations^1^, whom they eventually replaced. We assist to major behavioral changes with the gradual development of the cultural components of what would define the Upper Paleolithic and the cultural modernity^2^. Scarce during the European late Middle Paleolithic^3–6^, evidences of symbolic behavior exploded in term of quantity and diversity in the Early Upper Paleolithic (EUP) cultural material^7–11^. Significant technological advances were developed with the production of blades and notably bladelets in the Châtelperronian^12,13^, the intensification of the bladelet production with the Proto-Aurignacian and finally the individualization of their reduction sequence during the Early Aurignacian^14,15^. Simultaneously, craftsmen explored and mastered new raw material^10,16,17^, bones and teeth, producing a brand-new set of tools as a response to arising needs. The development of these new needs and subsequently these new bone technologies had direct consequences on the resources procurement and management strategies and, in a more general way, on the cultural relationships constructed by human with their animal counterpart. This is specifically attested by the development of figurative art, where mammals play a quasi-exclusive role during the EUP, and the introduction of mammal bones and teeth in the personal ornaments^10^, where previously only minerals and malacofauna were used^5^.

These symbolic and economic transformations of the societies occurred in a changing environment characterized by the major climatic shift of the MIS3^18^. In Southwestern France, the final Mousterian took place in a relatively temperate environment^19^ while the Upper Paleolithic is marked by a progressive climatic degradation that reached its acme with the Heinrich 4 event during the Early Aurignacian^20^. Woodland and steppic species were progressively replaced by arctic taxa^19,21,22^, reindeers becoming the base of the diet and a major raw material resource for human populations^16,22,23^.

Understanding the evolution of the subsistence strategies during such a period of environmental changes is of prime interest for apprehending the socio-economic transformations in the populations, which leaded to the numerous technical and symbolic innovations. In this context, Southwestern France occupies a particular place, being one of the few regions with a long detailed transitional period covering Late Mousterian, Châtelperronian, Proto-Aurignacian, and Early Aurignacian documented at numerous sites. In addition, numerous models on the hunting strategies evolution from the Middle to the Upper Paleolithic in Europe are at least in part based on south-west France^22–25^. However, since most of the sites yield only part of the transitional sequence in their stratigraphy, the studies generally focus on a specific section of the period^26^ or have to adopt a synthetic regional approach^23–25^. Yet, geographic variability can hamper large-scale zooarchaeological interpretations: local topography and micro-environmental variabilities condition the availability of resources in term of quantity and quality. Consequently, the absence of a specie in a faunal spectrum can sometimes only be related to local environmental parameters and the extent of its biotope and does not result from a hunting selection. As a result, the studies of the subsistence behaviors are directly impacted by environmental and seasonal parameters, and macroregional models must be tested at a site level to discriminate the geographical constraints from cultural shifts in the archaeological records.

Les Cottés (Saint-Pierre-de-Maillé, France) is one of the rare sites that offers, at the same location, every cultural units known for the transition from Middle to Upper Paleolithic in the area, including Châtelperronian and Proto-Aurignacian^27^ (Supplementary Information 1). Excavation at the site was resumed in 2006 and is still on-going with a multi-disciplinary team^28^. In turn, Les Cottés offers a unique opportunity to discuss the diachronic evolution of subsistence activities during this challenging transition. We propose here taphonomic and zooarchaeological analyses of the complete sequence of Les Cottés based on the recent excavations to explore the shift in technological, cultural and symbolic relationship between human communities and the macrofaunal component of their ecosystem. Our objectives are firstly to identify how the development of new technologies and inherent new needs might have influenced the evolution of the subsistence strategies, and secondly to discuss the consequence of this evolution in the human-carnivore competition for the access to the resource. In other words, we intend to establish how the change in the cultural and physical environment of the human populations during the transition from Middle to Upper Paleolithic modified the ecological contract between humans and nature.

**Figure 1:**
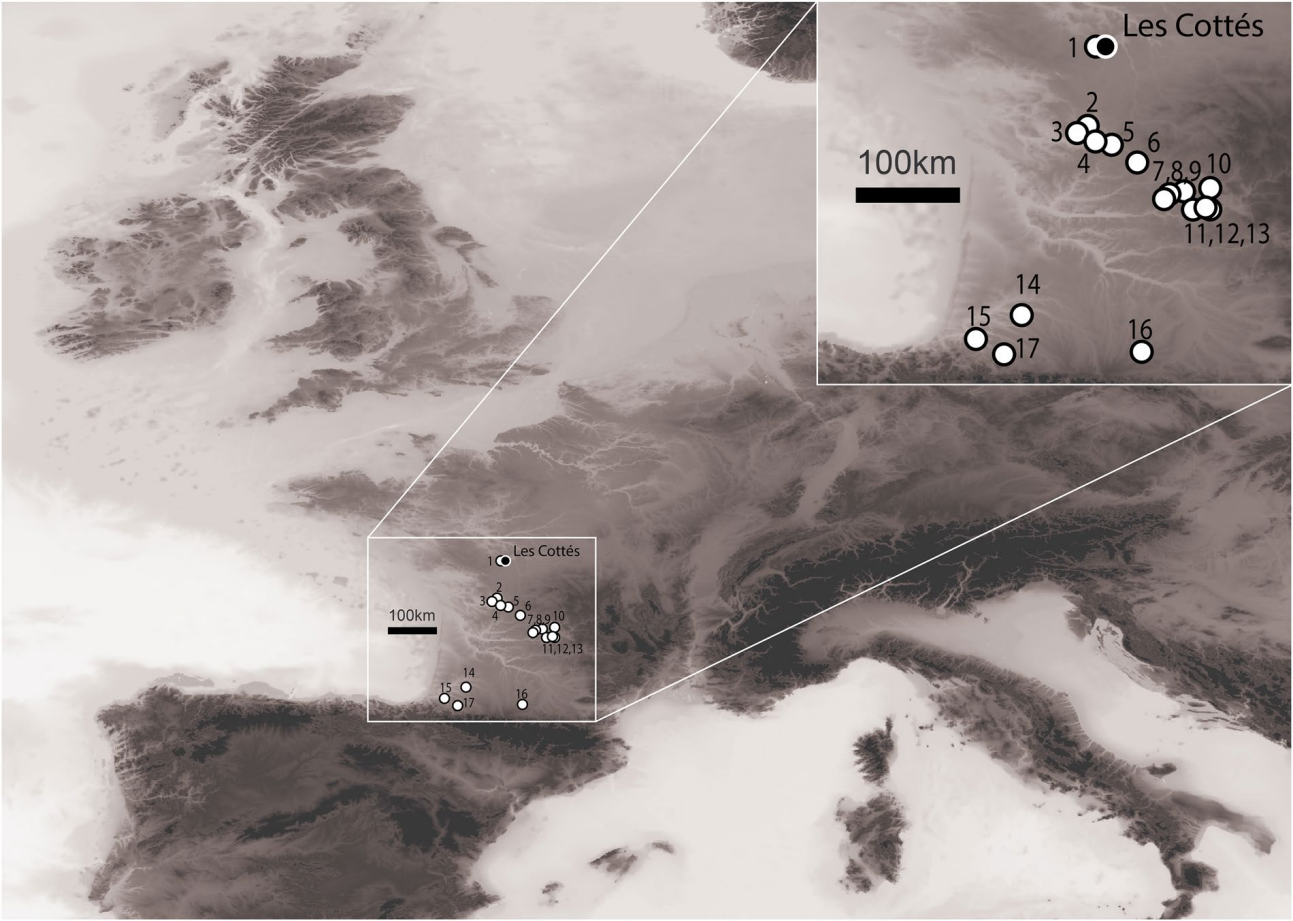
Distribution of the sites used for discussion. 1: Quincay; 2: Fontaury; 3: Les Rois; 4: La Quina Aval; 5: Trou de la Chèvre; 6: Les Battus; 7: La Ferrassie; 8: Pataud; 9: Castanet; 10: Les Battuts; 11: Grotte XVI; 12: Roc-de-Combes; 13: Le Piage; 14: Brassempouy; 15: Isturitz; 16: Les Abeilles; 17: Gatzarria. Map made by S. R. using the software QGIS 2.6.1 and Etopo1 Digital Elevation Mode.

Faunal spectra have been compared at a regional scale with 32 contemporaneous assemblages from 17 sites (Fig. 1) to evaluate how the climatic fluctuations impacted the prey availability and the evolution in the hunting selection. Skeletal part representations and carcass processing were considered for investigating how human hunting activities were inluenced by emergent needs for specific faunal raw materials. Finally, the relative contribution and impact on the assemblage of humans and carnivore was used as a proxy of their interactions and competition.

## Results

### Faunal spectra

Fragmentation and taphonomic events conducted to a limited proportion of identified remains (36%) (Supplementary Information 2). Most of the common ungulates known for that timeframe in Western Europe are documented in the deposits; reindeer, horse and bison are the most frequent species (Table 1). Carnivores progressively decrease through the Upper Paleolithic deposits.

**Table 1:** Faunal spectrum per stratigraphic unit (US). NR = Number of Remains.

	US02		US04UPPER		US04LOWER		US06		US08	
	NR	%NISP	NR	%NISP	NR	%NISP	NR	%NISP	NR	%NISP
Lagomorph		0%	2	0%	2	0%		0%		0%
Vulpine		0%	2	0%	4	1%		0%	4	1%
Wolf		0%		0%	1	0%		0%		0%
Hyena		0%	3	1%	7	1%	9	5%	4	1%
Coprolite		0%	1	0%	1	0%		0%	1	1%
Medium Size Carnivore	1	0%		0%		0%	1	1%		0%
Small Size Carnivore		0%	1	0%		0%		0%		0%
Large Size Carnivore		0%		0%		0%	1	1%	1	0%
Horse	5	1%	53	10%	137	22%	23	13%	32	11%
Hydruntinus		0%		0%	1	0%		0%	1	0%
Mammoth		0%	4	1%		0%	4	2%	4	1%
Capra		0%	1	0%		0%	1	1%		0%
Chamois	1	0%		0%		0%		0%		0%
Bison	7	1%	20	4%	43	7%	38	22%	105	37%
Rhinoceros		0%		0%	1	0%		0%	1	0%
Wild Boar		0%		0%	1	0%		0%		0%
Roe Deer		0%		0%	1	0%		0%		0%
Reindeer	426	97%	463	84%	436	69%	95	55%	127	45%
Red Deer		0%		0%		0%	1	1%	3	1%
Megaceros		0%		0%		0%		0%	1	0%
Nisp	**468**		**550**		**635**		**173**		**284**	
Identification Rate		**44%**		**38%**		**34%**		**38%**		**36%**
Small Size ungulate	5				2					
Meidum Size ungulate	205		283		280		44		60	
Large Size ungulate	48		95		231		89		230	
Megafauna			1						3	
NID	337		518		704		149		209	
Total	**1063**		**1447**		**1855**		**455**		**786**	

Large ungulates (Bison and Horse) are dominant in the Mousterian unit (US08: 48%). When taking together bison, horse and non-identified remains attributed to the “large ungulates”; the other units are dominated by the reindeer, whose percentage increase through the sequence ((US08: 45%; US06: 55%; US 04lower: 69%; US04upper: 84%; US02: 97%) (SI2 Table 4).

Reindeer falls within ‘medium size ungulate’ category, along with red deer, wild donkey, wild boar and ibex. However, out of 842 medium size ungulate identified bones, only 9 were attributed to another specie than reindeer (NISP red deer = 4; NISP wild donkey = 2; NISP wild boar = 1; NISP ibex = 2) suggesting that the overwhelming majority of medium size ungulate bones belongs to reindeer.

Megafauna is extremely rare in the Upper Paleolithic units, with only one rhino tooth in US04*lower* and fragments of mammoth molar in US04*upper*.

A taxonomic analysis using mass Spectrometry analysis of collagen proteins has been used to identify previously unidentified remains^29^ (SI2 Table 5). While it did not change significantly the faunal spectra, it helped to identify some rare taxa (such as rhinoceros in US2, wolf in US04upper).

### Origin of the accumulation

While carnivore modifications are extremely limited in US02 (0.5%), 04*upper* (2.6%) and 04*lower* (4.5%), they concern more than 10% of the remains of the Chatelperronian and Mousterian layers (SI2 Table 6 and 7). These modifications are mostly centered on carnivore remains and large ungulates ones (8.48%NISPLargeUngulates and 26.09%NISPCarnivore). Reindeer remains are relatively more spared compare to the ones of larger species in the Upper Paleolithic layers. The same appears to be true for the lowest stratigraphic units, but the difference is not statistically significant (US04upper: Khi^2^ = 7.224, p < 0.01; US04lower: Khi^2^ = 10.064, p ≪ 0.001; US06: Khi^2^ = 1.71, p > 0.05; US08: Khi^2^ = 3.381, p > 0.05). Carnivore damage stays relatively limited and is always lower that the one identified on carnivore sites and coherent with their secondary access to carcasses (SI2 Fig. 1). Thus, carnivore played very limited roles in the accumulation of small and medium size ungulates during the whole sequence deposit and, potentially, only a moderate one for the accumulation of large size ungulates during US 06 and US 08 deposit.

Frequencies of remains exhibiting anthropic marks (cut-marks, notches, burnt bones, scrapping marks, bone tools) ranges between 22% (US06) and 34% (US04upper) (Table 2) and are comparable to what is observed on contemporaneous human accumulations^22^. Except for the megafauna of US08 (SI2 Table 8), all species are concerned by these modifications.

**Table 2:** Anthropic modifications per layer. NRanthro: bones exhibiting human modification.

		US02	US04*upper*	US04*lower*	US06	US08
	NRa	765	970	1160	403	671
NRanthro	**NR**	**191**	**251**	**394**	**90**	**182**
	**NR%**	**24.97%**	**25.88%**	**33.97%**	**22.33%**	**27.12%**
Cut-marks	**NR**	99	138	119	42	83
	**NR%**	**12.94%**	**14.23%**	**10.26%**	**10.42%**	**12.37%**
Scrapping	**NR**	16	39	15	6	11
	**NR%**	**2.09%**	**4.02%**	**1.29%**	**1.49%**	**1.64%**
Retoucher	**NR**	6	9	3	3	8
	**NR%**	**0.78%**	**0.93%**	**0.26%**	**0.74%**	**1.19%**
Notches	**NR**	44	60	88	17	28
	**NR%**	**5.75%**	**6.19%**	**7.59%**	**4.22%**	**4.17%**
Bone flakes	**NR**	8	11	20	6	37
	**NR%**	**1.05%**	**1.13%**	**1.72%**	**1.49%**	**5.51%**
Burned bones	**NR**	37	61	179	32	26
	**NR%**	**4.84%**	**6.29%**	**15.43%**	**7.94%**	**3.87%**

Expressed in NR and %NR. NRa = Number on analyzed remains. The percentages were calculated on observable remains only.

### Human carnivore interactions

The human-carnivore interactions were complexes and varied through time at Les Cottés. In US 08, the time separating men and carnivore occupations was probably very limited since at least seven bones bear both human and carnivore marks. In one case, the superposition of a tooth mark over a cut-mark attests of the anteriority of the second. One of the pieces Y5-2958 is a bison tibia shaft fragment used as retoucher, that also exhibits pit marks and was chewed by a large carnivore. Otherwise, in US 06, US 04*uppe*r and US 02, six carnivore remains, show cut-marks that attest of their exploitation by the human populations. Notably, in US06 a Hyena phalanx bears a cut-mark on its palmar surface.

### Reindeer skeletal profile

For all levels, skeletal profiles show an underrepresentation of the axial parts (at the exception of antler and teeth) toward the appendicular skeleton (SI3-Table 1). The scarcity of ribs (NISP = 87; MAU = 0.5) is not only linked to a fragmentation or identification bias since only 64 unidentified medium size ungulate remains were attributed to flat bones for the whole sequence while 842 were attributed to long bones. This confirms the over-representation of appendicular bones on the assemblages.

Comparisons of skeletal element frequencies to bone density values for reindeer show a weak but positive significant relationship, indicating that the less dense elements are less frequent than the densest ones. (Si3 Fig. 1). It is also illustrated by the ratio of long bones extremity remains (NISPextremities/NISPlongbones US 02 = 6/253; US 04*upper* = 7/226; US 04*lower* = 9/283; US 06 = 4/46; US 08 = 6/69), known to be more easily altered. The heterogeneity in the skeletal representation observed can not be only due to differential preservation, as dense parts, like carpals and tarsals are scarce while they have a density index comparable to the one of some long bone portions well represented in the assemblage. Also, US 02 and US 04*upper* are characterized by an abundance of antler remains, largely more common than other denser elements. Especially, they are twice more frequent than teeth remains in US 02.

Evaluation of reindeer skeletal part representation suggests that differential conservation cannot explain the whole variance of the profiles and that other mechanisms were at stake when it comes to the incompleteness of carcasses found at the site. Another explanation is that the missing skeletal elements were not introduced into the site due to a selective transport linked to the nutritive value of the different elements, but this hypothesis is not very pertinent for carpals and tarsals bones, as they are always less frequent than adjacent long bones.

**Figure 2:**
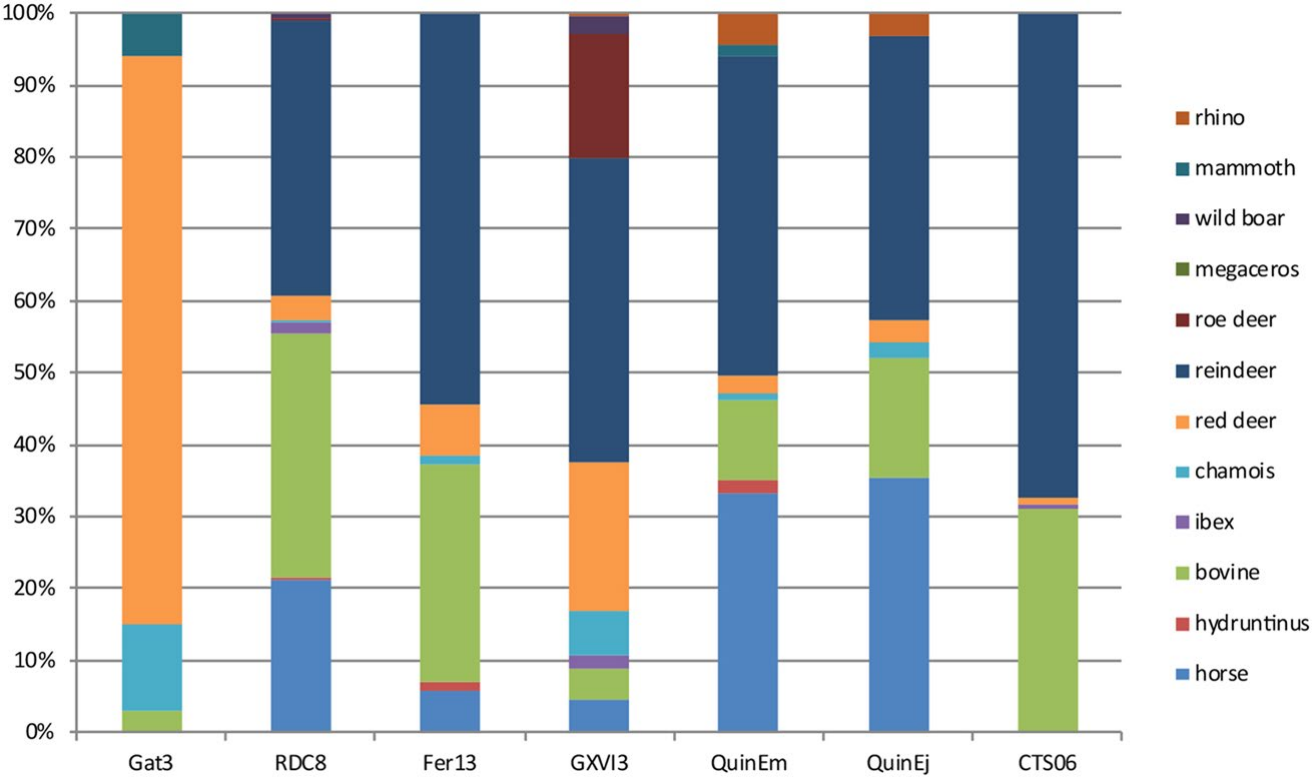
US06 faunal spectrum (on the right part of the graph) compared to other Chatelperronien assemblages from the same region. QuinEm = Quincay, l. EM^66^; RDC8: Roc de Combe l. 8^26^; Fer13:La Ferrassie l.13^67^; Gat3: Gatzarria l. 3^66^; GXVI3: Grotte XVI^68^; QuinEj = Quincay, l. EJ^66^. See also^19^.

While there is no correlation between skeletal part frequencies and FUI^30^ (i.e. no evidence of selective transport based on the meat value of the elements), there is a strong and highly statistically significant relation with the quantity and quality of the marrow contained in the different elements, whatever the Oleic Acid index^31^ or the MUI^32^ is considered (SI3 Figs 1 and 2).

For all the sequence, we can see that same selective transport strategies were undertaken by the human groups, whatever their techno-culture and under different environmental constraints, favoring the gathering of bones rich in marrow and the introduction within the site of incomplete carcasses. The elements with the lowest food values (carpal, tarsal, part of the skull etc.) would have been discarded at the kill site after the conduction of a first butchery. The selective introduction of some skeletal parts into Les Cottés is a strong evidence that the site was used all along as a consumption site. Within this common pattern, some minor variations exist (e.g.: the relative abundance of antler remains in the upper most units) that might find an origin within the carcass exploitation and the need for early upper Paleolithic populations for some specific raw materials (see below).

### Carcass exploitation

Due to his high frequency in each layer and its exclusive human origin, only reindeer is adapted to a diachronic approach of the carcass processing. Cut-marks were observed on all reindeer skeletal elements for each level, but variations exist in their frequency. The frequencies have been calculated and compared on medium size ungulate remains (reindeer + unidentified medium size ungulate remains) preserving at least 50% of their cortical surface. US 04*upper* shows a higher rate of cut-marks on medium size mammals. When preservation of cortical surface is taken into account, the difference with US 02, US 06 and US 08 nearly disappears (respectively: Khi^2^ = 0.066; Khi^2^ = 0.799; Khi^2^ = 1.31299) but it is still statistically significant with US 04*lower* (Khi^2^ = 287.18; p << 0.001). While for every level dismembering, skinning and defleshing activities are attested, the analysis of cut-marks distributions on long bones testify of changes in the carcass processing and on the nature of the resources looked for^33^. For example, the circular incision of the skin is documented on metapodial fragments on each layer; however, this has been performed at the distal shaft for US 08 and mid- shaft for other layers. Moreover, for US 02, two phalanges (first phalange and one vestigial) exhibit transversal cut-marks, confirming that circular incisions were alos made on autopodial bones. For this layer, a longitudinal incision let also its mark on the lateral side of a metapodial. Incompatible with the will to recover the skin in its larger dimension^22^, it could be interpreted as (1) either the absence of interest for the skin of the legs or (2) a multiple step process, beginning with the removal of the skin related to the defleshing and followed by a distinct processing of the lower limb skin. The identification, on one faunal remain, of a circular incision in the mid-shaft associated with short longitudinal incisions below supports the second hypothesis. Defleshing lets mostly marks on the meaty long bones and is particularly visible on US 04*upper* where 74% of the femur remains exhibit defleshing cut marks whereas they are only visible on 31%of US 04*lower* and 47% of US 02 femur remains. In US 08, defleshing is notably identified on one of the only two humerus fragments, which bares numerous longitudinal cut marks that might suggest the recovery of fillets for the confection of dried meat^34^, but other evidences are needed to sustain this hypothesis and to go any further. Finally, one of the most interesting results comes from the analysis of metacarpal anterior grooves. The high frequency of marks in US 04*upper* (10/19 in NISP) attesting of the recovery of sinews diverges with the ones observed on the other stratigraphic units (number of occurrences of sinew extraction on metacarpal remains: US 02 = 1/11, US 04*lower* = 1/23, US 06 = 0/2). Thus, it seems that this specific faunal resource was systematically looked for during the Early Aurignacian occupation.

US 04*upper* and US 02 present a large quantity of antler remains. The presence at the site of more than a dozen of split-based points^35^, but also evidences of antler’s exploitation (sawing, debitage, etc) as well as the discovery of a partial form of spear point strongly suggest that antler remains result from the acquisition and or the production of blanks for the latter production of spear points. In at least two cases in US 04*upper*, the connections of antlers to the frontal bone attest that part of their acquisition at least was directly integrated to the hunting activities rather than resulting from the collect of shed antlers.

Beside reindeer resources, the Early Aurignacian deposit (US 04*upper*) shows also the exploitation of new categories of faunal raw materials for symbolic purposes. Thus the accumulation of mammoth dentine ivory remains (NISP = 4) can be put in relation with the presence in the site of personal ornament made in such material^36^. Since it is commonly accepted that such beads are made with fossil dentin^37^ and since no other mammoth elements were found in the same layer, it is reasonable to assume that their accumulation did not result from hunting activities.

Finally, carnivore exploitation is evidenced in post Middle Paleolithic deposits. In US 06, a hyena first phalange (W6-99) shows an incision on its plantar side coherent with skinning. In layer 04*upper*, two fox remains—a tibia distal extremity (S4-64) and a calcaneus (W8-336)—bear cut-marks that can be considered as evidence of skin recovery^38^. In addition, a recent study of the personal ornaments has identified two drilled fox canines at different stages of production confirming the *in situ* production of beads^36^. Associated with the cut-marked fox remains, this sustains the hypothesis of an integrate acquisition of these canines to the subsistence activities.

### Result summary

US 08 is dominated by large ungulates. The assemblage has mostly a human origin but carnivore also contributed to its formation. As for all the others units, there is a selective introduction of the elements rich in marrow. US 06: reindeer became the main prey; even if carnivores are quite frequent less remains show damages than can be related to them. Some of their remains even indicate that humans exploited them. US 04lower: reindeer frequency increased drastically in the faunal spectrum. The importance of carnivore damages almost disappeared from the assemblages. US 04lower and US 02: the faunal spectrum is specialized on reindeer. Carnivores were used for food and personal ornaments. Antlers seem to have been selectively introduced to the site for the preparation of spear points and bone tool industry. Some specific raw material (tendons) were systematically retrieved.

## Discussion

**Figure 3:**
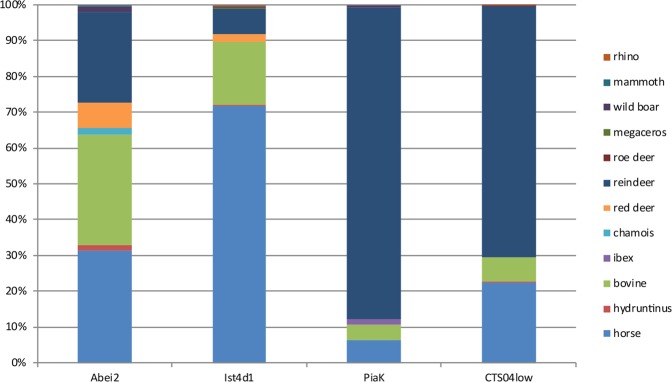
US04 lower faunal spectrum (on the right part of the graph) compared to other Proto-aurignacian assemblages from the same region. Abei2: Les Abeilles l.2^22^; Ist4d1: Isturitz l. 4D1^22^; PiaK = Piage l.K^69^. See also^19^.

**Figure 4:**
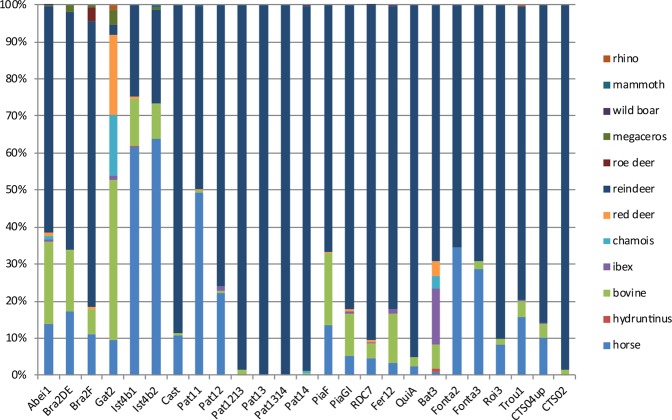
US04 upper and US02 faunal spectra (on the right part of the graph) compared to other Early Aurignacian assemblages from the same region. Abei1: Les Abeilles l.1^22^; Bra2DE & Brad2DF: Brassempouy l. 2DE & 2F^70^; Cast: Castanet^71^; Gat2: Gatzarria l. 2^67^; Ist4b1 + Ist4b2: Isturitz l. 4b1 and 4b2^22^; Pat11 + Pat12 + Pat13 + Pat1314 + Pat14: Pataud, l. 12, 13, 13–14 and 14^72,73^; PiaF + PiaGI = Piage l. F and Gl^70^;QuiA: La Quina Aval^74^; RDC7: Roc de Combe l. 7^26^; Bat3: Les Battuts l. 3^75^; Fer12:La Ferrassie l.12^68^; Fonta2 + Fonta3: Fontaury l. 2 and 3^67^; Roi3: Les Roi l.3^76^; Trou1: Trou de la Chèvre l.1^77^; AbChB:Abri du Chasseur^78^. See also^19^.

**Figure 5:**
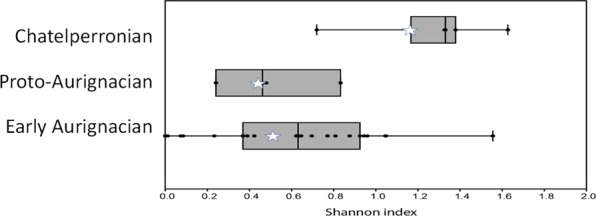
Shannon diversity index distribution for the different techno-complexes (see SI2 for details). The stars position Les Cottés assemblages.

The faunal spectra highlight an evolution in the hunted preys, with a steady increase of the reindeer in the human diet. It is always difficult to establish to what extent faunal spectra reflect the environmental availability of resources rather than hunters selection strategies^23,39^. Comparison to contemporaneous data can at least identify if we are confronted to a local or a regional pattern. Characterized by a human deposit modified by carnivore activities, Les Cottés US08 is excluded from the comparison. Although, Les Cottés is localized in the northern-most margin of the sites that yielded faunal spectrum for this period, the ungulates faunal spectra share many similarities with the contemporaneous ones from the Aquitaine basin and the Pyrenees (Figs 2–4). The progressive increase of reindeer through the stratigraphy at les Cottés can be observed at a regional scale and thus do not result from local conditions, as it has already been established elsewhere^23^. However, Les Cottés stands out by the relative low diversity of its spectra: as measured using the Shannon diversity index, the faunal diversity is always in the lower range of the variability for the different culture (Fig. 5). This specificity could be linked to the larger diversity of environments in the core of the Aquitaine Basin, often presented as a refuge-zone^40^.

Large ungulates are progressively replaced by the reindeer during the Middle to Upper Paleolithic transition^19^. The relationship between this change in faunal spectra and reindeer hunting specialization by AMH has been largely debated during the late 90’s and the early 2000 years^23,39^. However, it is now accepted that this change resulted mostly from a global cooling during the second part of MIS3 when groups of AMH settled into Europe^19,20^. Banks and colleagues^20^ proposed—based on a synthesis of radiometric dates using Bayesian modelling—that Early Aurignacian deposits in Southwestern France should be attributed to the cold Heinrich 4 event. At les Cottés, the reindeer increase reaches its acme in the Early Aurignacian stratigraphic units (US 04*upper* and US 02), which dates are compatible with the timeframe of Heinrich 4 event^41,42^. Temperate species (red and roe deer, wild boar), that are present from the Middle Paleolithic to the Proto-Aurignacian deposits, were replaced by mountainous species (ibex, chamois in US 04*upper* and US 02). The presence of the latter in a region of low altitude would have been linked to the development of altitude ice sheets and an important decrease of temperature in low altitude areas. Thus, these elements are coherent with a major cooling of the environment. A recent analysis of clays diagenesis throughout Les Cottés’ sequence sustains such climatic pejoration^43^. The stratigraphy here offers one of the rare examples of a detailed continuous record of major pejorative climatic conditions occurring during late MIS3. In addition, based on their similarity with other regional contemporaneous sites and their relation with the climatic contexts, it seems that the spectra of Les Cottés directly reflect the environmental conditions, with limited or no distortion due to hunting selection in terms of species procurement. Consequently, it implies that during the whole sequence the different human populations applied the same hunting strategy by selecting the most common taxa in their territory, which appear to be also the highest ranked resources available in the environment.

Les Cottés does not only inform us on the relation between human and their animal environments but also on the dialectic that slowly developed between environment shifts, new needs and the technological innovations.

Some of the predation behaviors show some diachronic consistency at the site, suggesting that they were not directly dependent of cultural factors. The most common species in the environment which appeared to be the highest ranked resources were hunted: species found at Les Cottés are found in the comparable proportion in other contemporaneous sites^19,22^.

The selective transport of the carcasses follows all along the deposits the same criteria: the relative richness in marrow. The landscape of Les Cottés is characterized by limestone plateaus cut by large open valleys, offering favorable conditions for reindeers, horses and bisons, which would have easily grassed in small group all around^44^. In addition, the implantation of the occupations was not only selected for hunting purposes but probably also for other reasons like maybe the quality of the lithic raw materials available in the area^45^ and the characteristics of the shelter.

The exploitation of animal raw material (both alimentary and technical) was systematically conducted on the site attesting of its used as a processing and consumption site throughout time. On the opposite, several differences in the most recent deposits suggest some cultural shift in the subsistence activities undertaken at the site, which are more easily understood in the light of the analyses of the other cultural materials. Little information is available for US 08 and 06 due to the relative scarcity of material (US06) and the contamination of the human accumulations by carnivores (US08). Only can we advance that the high quantity of carnivore marks on the faunal remains, even on the one bearing cut-marks, attest of their repetitive frequentation of the site and thus that the human occupations were probably relatively short and repetitive.

In US 04*lower*, preys with different behaviors were hunted, certainly during different hunting events instead of a mass killing. The study of the lithic industry has demonstrated that domestic and cynegetic personal gears were produced on-site^27^. High velocity impact fractures on bladelets indicate their use as armature and that they might have been re-introduced in the site while they were still blocked inside the carcasses brought at Les Cottés. The exploitation was conducted *in situ* and parts of the bone wastes were then burned in hearts (that were not discovered during the excavation). These zooarchaeological proxies, the small impact of carnivores and the thickness and richness of the layer point toward numerous occupations and the use of the site as a residential base camp. Carnivores had continued to come sporadically at the site between the passages of the men.

US 04*upper* faunal spectrum is characterized by the reindeer dominance. Like in the previous layers, carcasses were introduced incompletes within the site, with a strong selection of the elements reach in marrow. They were intensively butchered and all usable products were looked for. Even sinews and antlers were intensively collected. Some of the beads were produced *in situ*^37^. AMH appear to have invested a great amount of time and efforts in processing the non-nutritional faunal raw material (reindeer antler, skin, sinew, fox tooth, mammoth ivory). The recent lithic analysis confirms the high quantity of standardized scraper produced and used on the site^27^, sustaining that this materials were processed at Les Cottés. Blanks for personal ornaments and bone tools might have been introduced following two different processes. Only found in fragments of small dimensions, mammoth ivory blanks were probably imported already pre-shaped within the site. On the opposite, fox canines and deer antlers might have been acquired directly through hunting. Les Cottés site was used as a place of production, certainly over a long period considering the diversity of activities undertaken there. Compared to the previous occupations, not only numerous activities were undertaken, but some of the activities would have necessitated a certain amount of time to be performed properly. At that point, carnivore had stopped to visit the site.

Finally, subsistence strategies conducted on the latter Aurignacian deposit—US 02—do not significantly differ from the one of US04*upper*.

Concomitant to the change in the hunted preys and the relative increase of the reindeer presence within Les Cottés environment, the activity of carnivores and the evolution of their relation with humans changed drastically between US 08 and US 02. The progressive decrease of their impact on the assemblages is observed. Furthermore, the nature of human-carnivore interactions evolves drastically through time at les Cottés. In US 08, 7 remains show modifications caused by the two taphonomic agents, including the tibia bone used as retoucher. Our results suggest that the time separating the occupations by the two accumulators was short enough so that the bones, discarded by the men, conserve nutritive value for the hyenas when they came for scavenging. On the opposite, during the US 06 deposit, carnivores continued to come at the site when humans were away but the mark on the hyena first phalange (W6-99) suggest that carnivores had begun to be incorporated to the subsistence economy.

During US 04*upper* deposits, the exploitation of fox fur and meat shows a certain consistency with what is known on the Early Aurignacian small carnivore exploitation^22^. Here, after hyena disappeared from the region, a new carnivore was incorporate to the economy. Simultaneously, intrusion of carnivores in the material culture of the Early Aurignacian—i.e. the perforated canines^37^—is an evidence for their potential role in social interactions.

Thus, the frequency of the interactions between human and carnivores and their nature seem to have evolved at the site following the need for new raw materials and equipment. Like for the demise of large carnivores, the increase of small carnivore use for technical purpose is a common trait for most of the Early Aurignacian site of the region^26,46^. Discamps^47^ underlined the decreasing of carnivore activities in assemblages from southwestern Europe all along MIS 3, resulting in the nearly disappearance of Hyena from the whole region at the end of the Aurignacian era, a phenomenon that might be related to the increasing predatory pressure of human societies on their environment leaving nearly no place for competitors (see Stiner and Khun^48^ for a discussion of the same phenomena in the Mediterranean basin). The development of brand-new sets of weapons (lithic bladelet production and antler spear point)^14,16^ would have allowed the production of lighter spears and potentially longer-range weapons, increasing the hunting calorie return^22^.

At the same time, based notably on the augmentation of the number of sites through time, an increase of human population at the dawn of the Upper Paleolithic is also frequently suggested^49,50^ (contra)^51^ which also might have conducted to an increase of the human pressure on their biotope. The development and generalization of personal ornamentation^10,38^ related to group identity materialization may also be related to population density increase. Simultaneously, developments of the acquisition raw material networks (lithic, personal ornament etc: i.e.^10,14^) suggests a stronger structuration of the social landscape and a territorialization of the economy.

In sum, if Les Cottés has always been used as a campsite, the number of activities undertaken there and the duration of site occupation evolved. These changes in the subsistence activities evolved in straight correlation with the needs for new raw materials, a constraint resulting from the innovation of the Upper Paleolithic in term of lithic technology, bone industry and symbols. At Les Cottés, cultural changes are not seen in the hunting strategies, since the same prey selection and the same selective transport were carried out throughout the stratigraphy, but rather in how resources were processed and incorporated in the subsistence and symbolic economy.

As it has been demonstrated with the economy of mammal hard tissues^16^, taxa were incorporated at different levels in the economic life of human societies. Species remains were not used for the same purposes: Mammoth ivory was shaped as figurine or flutes^7^ such as bird bones; mammoth and carnivore teeth were used for personal ornaments^10,38^; ungulate bones for bone industry and use for domestic work and only deer antlers were transformed for weaponry^52^. Some taxa were only used for symbolic means and specific skills were developed to transform this large diversity of materials^53^. In the same way the evolution of the human-carnivore relation during the Middle to Upper Paleolithic transition, where carnivore status changed from competitor into preys and symbols, attests of the new perception that human had for their animal environment, which begun to be thought and symbolized. The faunal environment seems to have found a specific place within the cosmogony of the Upper Paleolithic as its omnipresence in the figurative arts is attested as early as the Early Aurignacian on parietal art^54^. This incorporation of animals within the social and economic life of past human society was a constant during the whole Upper Paleolithic.

## Method

All the remains were observed by the same analyst (WR) and were double-checked by at least one of the two others (SyR, MCS). For taxonomic and anatomical identifications, we used the reference skeletal collections from the the PACEA Laboratory (CNRS- Bordeaux University -MCC) and the TRACES Laboratory (CNRS-Toulouse Jean Jaurès University) and punctually the *Virtual Faunal Comparative Collection* from the Max Planck Institute^55^. Pieces were identified at the most precise level and, when it was not possible to propose a specific attribution, ungulate size classes were used^56^. With regard to the skeletal part profiles, all identifiable specimens (including shaft fragments) were taken into account and recorded following the “element, portion, segment”^57^. Shaft fragmentation was evaluated using the Shaft length and Shaft circumference indexes^58^. Analyses of the bone surfaces were conducted on all the identified remains and part of the non-identified ones. Bone surfaces were observed under a low-angled light using a hand lens (enlargement: 10x) for the taphonomic and zooarchaeological observations. Weathering, root etching, anthropogenic and carnivore modifications were systematically looked for^59–63^. Oxide colorations of the bone cortical surfaces were also recorded. The proportion of preserved cortical surface was estimated per quartile^64^. When unclear modifications were detected, specimens were subjected to more thorough evaluation with a 20–80x microscope. Percentage values were calculated based on the number of analyzed remains (NRa). Bones with unobservable surfaces were excluded for the calculation of the percentages of modified bones. NRa can change depending of the analysis type. Skeletal part representations were established for the reindeer (only taxa exhibiting more than 100 identified remains and for which the human origin has been demonstrated) using MAU index. Differential preservation has been tested by confronting frequencies of skeletal elements and their respective densities^65^. Possibility of a selective transport based on the nutritive value of the elements was tested using notably the SFUI^31^ on the reindeer, the marrow cavity volume of bones^33^ and the oleic acid index UMI^32^. We have evaluated the results statistically using Spearman’s rank correlation (r_s_). Each faunal remain that could be replaced precisely on a complete bone was recorded on bone templates in *Adobe Illustrator*. The cut-marks were then interpreted using a recently published cut-marks coding^66^.

## Supplementary information

Sypplementary information file

## Data Availability

All data generated or analyzed during this study are included in this published article (and its Supplementary Information files).
