# Supplementary material for: Subsistence strategy changes during the Middle to Upper Paleolithic transition reveals specific adaptations of Human Populations to their environment
Source: Sci Rep. 2019 Nov 1;9:15817. doi: 10.1038/s41598-019-50647-6 (PMC6825241; doi:10.1038/s41598-019-50647-6)
Supplement: Supplementary file 1 — Sypplementary information file [file 41598_2019_50647_MOESM1_ESM.docx]

**TITLE: “Subsistence strategy changes during the Middle to Upper Paleolithic transition reveals specific adaptations of Human Populations to their environment”**

William Rendu*^1,2^, Sylvain Renou^1^, Marie-Cécile Soulier^3^, Solange Rigaud^1^, Morgan Roussel^4^, Marie Soressi^4,5^

1 : PACEA, UMR 5199, CNRS, Université de Bordeaux, Ministère de la Culture et de la Communication (MCC), F-33400

Pessac, France

2 : New York University, Department of Anthropology, CSHO, New York, NY 10003, USA

3 : TRACES, UMR 5608, CNRS, Université Toulouse Le Mirail, F-31058 Toulouse Cedex 9, France

4: Faculty of Archaeology, Leiden University, 2333CC Leiden, Netherlands

5: Dept. of Human Evolution, Max Planck Institute for Evolutionary Anthropology, Deutscher Platz 6, 04103 Leipzig, Germany

**** Corresponding Author***: William RENDU [wrendu@u-bordeaux.fr](mailto:wrendu@u-bordeaux.fr). Phone : +33 651 98 3308

## **Supplementary Information**

## Supplementary information 1

**Les Cottés site**

Discovered in the late nineteenth century^1^, les Cottés (Saint-Pierre-de-Maillé, France) is a small limestone cave located in the southwestern part of the Parisian Basin in France.


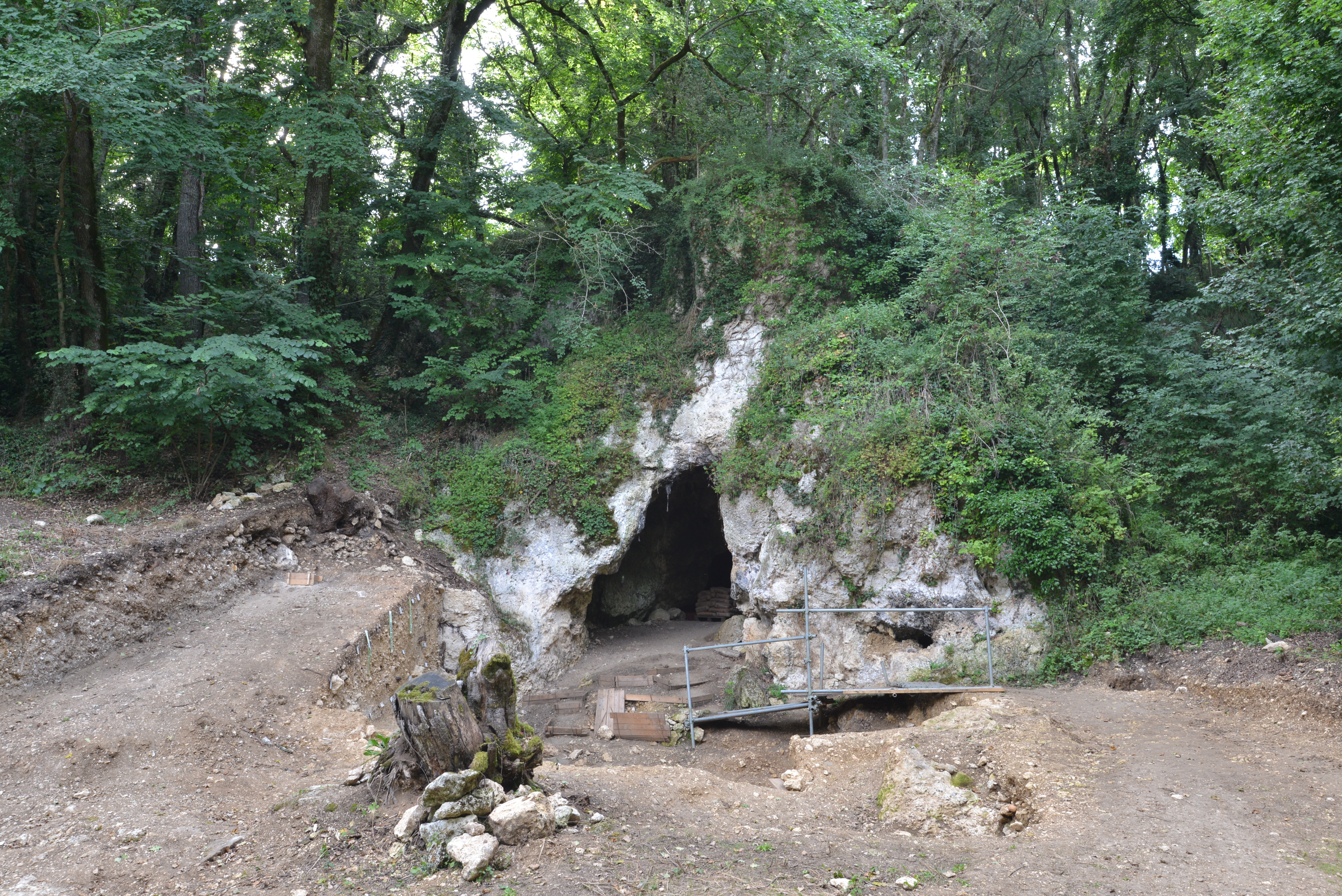


**SI1 Figure 1. Les Cottés site view from the East. Photo from Les Cottés Project.**

A diamicton of mostly centimeter-sized limestone clasts and a sandy clay matrix preserve Late Middle and Early Upper Paleolithic occupations in the entrance of the cave. During the 60’s Pradel^2^ defined a sequence of Mousterian, Chatelperronian, Early Aurignacian and Gravettian layers at the site. The faunal stock excavated by Pradel was analyzed by Bouchud ^2^who provided the first quantified faunal spectra for the sites (**SI1 Table 1**)

|  | **Reindeer** | **Bovins** | **Horse** | **Red deer** | **Capra ibex** | **Cave lion** | **Wolf** | **Hyena** | **bear** | **Rhinoceros** | **Mammouth** |
| --- | --- | --- | --- | --- | --- | --- | --- | --- | --- | --- | --- |
| **Mousterian** | 2 | 15 | 9 |  |  |  |  | 1 | 1 | 1 | 1 |
| **ChatelpErroniAn** | 2 | 4 | 8 |  |  | 1 |  | 1 | 1 | 1 |  |
| **Aurignacian** | 5 | 3 | 10 | 2 | 1 |  | 1 | 1 | 1 | 1 |  |

**SI1 Table 1** **Faunal spectra from the Pradel excavation, after Bouchud 1961.**

Since 2006, Les Cottés has been the subject of a general recovery by an interdisciplinary field team and a new stratigraphic sequence with new dating was obtained.


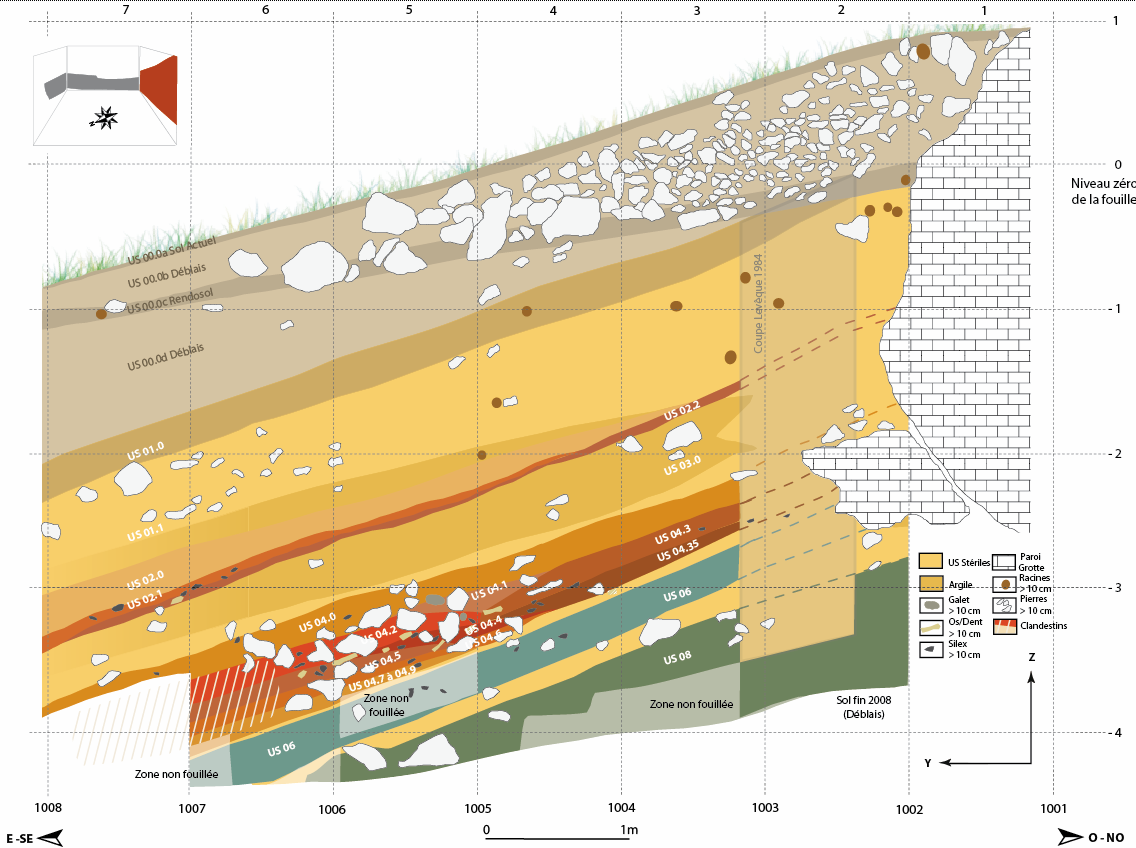


**SI1 Figure2: Stratigraphy of Les Cottés South profiles after (Soressi et al., 2010). US 04upper is constituted by US04.0, US04.1 and US 04.2. US 04lower is constituted by the other US04 subdivisions (see Soressi et al., 2010). Graphic . from Les Cottés Project.**

The sequence contains five major stratigraphic units: one Mousterian (US08), one Châtelperronian (US06), one Proto-Aurignacian (US04lower) and two Early Aurignacian (US04upper and US02). The Mousterian and Chatelperronian at Les Cottés were separated from the Proto-Aurignacian layers with a very low density deposit that spans approximately 1,000 years^3^. Radiometric dates on bones and on sediment indicate that the site was occupied from at least 45,000 years ago up until around 35,000 years ago^4,5^. The excavated area is of 15m^2^ and yielded a large stock of faunal and lithic remains. All fauna remains larger than 30 mm were given 3D coordinates with a total station. Identifiable bones and every macrofaunal tooth fragment smaller than 30 mm were systematically collected. The coarse fraction (>4 mm) coming from water sieving of the sediment was sorted to separate lithic, fauna and any other archaeological items. All identifiable faunal remains were then added to the faunal stock. In 2008, A human tooth was discovered and was recently attributed to the Neanderthal taxon based on its genomic sequence^6^.

## Supplementary information 2

**Taphonomic characterization of Les Cottés assemblages**

The taphonomic modifications are variable from one layer to another (SI2 table1). Weathering is common on the assemblages but is more abundant in US 06. At the same time, a large proportion of the remains from US 06 is smoothed suggesting possible movement within the sediment after their deposit.

|  |  | **US 02** | **US 04upper** | **US 04lower** | **US 06** | **US 08** |
| --- | --- | --- | --- | --- | --- | --- |
|  | **NRa** | **794** | **829** | **1051** | **300** | **620** |
| **Cracks** | **NR** | 160 | 192 | 349 | 112 | 202 |
|  | **%NR** | 20,2% | 23,2% | 33,2% | 37,3% | 32,6% |
| **Exfoliation** | **NR** | 305 | 310 | 396 | 140 | 184 |
|  | **%NR** | 38,4% | 37,4% | 37,7% | 46,7% | 29,7% |
| **Frost modification** | **NR** | 75 | 121 | 123 | 74 | 91 |
|  | **%NR** | 9,4% | 14,6% | 11,7% | 24,7% | 14,7% |
| **Rounded bones** | **NR** | 60 | 75 | 53 | 49 | 19 |
|  | **%NR** | 7,6% | 9,0% | 5,0% | 16,3% | 3,1% |
| **Fero Maganese spots** | **NR** | 202 | 243 | 607 | 166 | 247 |
|  | **%NR** | 25,4% | 29,3% | 57,8% | 55,3% | 39,8% |
| **Fero Maganese covering** | **NR** | 411 | 17 | 40 | 5 | 8 |
|  | **%NR** | 51,8% | 2,1% | 3,8% | 1,7% | 1,3% |

**SI2 table 1: Principal non-biologic taphonomic modifications of the faunal stock**

Dark Ferro-Manganese deposits are focused on US 02 and US 04*lower*, where more than 50% of the remains exhibit such coloration. In layer 04*upper*, bones are largely colored by ochre deposits (34%NRa) while this modification is nearly absent from the other assemblages), making it a useful stratigraphic marker (**Erreur ! Source du renvoi introuvable.**)^7^. It can be directly linked to the abundance of oxide within the sediment of the level, which was characterized by an intense red color in the field^3^. This coloration has no impact on the cortical surface analyses. This feature is highly common in Early Aurignacian deposits and is often interpreted as resulting from the intense ochre work during the occupations^8^.

|  | **US02** | **US04upper** | **US04lower** | **US06** | **US08** |
| --- | --- | --- | --- | --- | --- |
| NRa | **256** | **393** | **640** | **266** | **376** |
| NR | 0 | 106 | 5 | 1 | 0 |
| %NR | 0,0% | 27,0% | 0,8% | 0,4% | 0,0% |

**SI2 Table2 : Frequency of ochred bones. The analysis was performed on a sample of the different assemblages.**

Bone preservation is globally good except for US 06 where 46% of the remains retained less than half of their cortical surfaces, limiting the recognition of modifications such as human cut and carnivore tooth marks (SI2 table3). Considering the taphonomic difference between the layers and since some of the modifications appeared during or just after the deposition of the remains, we can assume that if some displacements had occurred, they were likely limited.

| **% stage of cortical preservation** | **US02** | **US04UPPER** | **US04lower** | **US06** | **US08** |
| --- | --- | --- | --- | --- | --- |
| >75% | 44,22% | 61,13% | 55,56% | 41,50% | 52,66% |
| >50% | 23,95% | 13,45% | 16,17% | 12,50% | 16,03% |
| >25% | 13,06% | 9,49% | 8,58% | 13,00% | 10,87% |
| >0% | 18,78% | 15,93% | 19,69% | 33,00% | 20,43% |
| NRa | **735** | **885** | **1107** | **400** | **671** |

**SI2 Table3: Preservation of the cortical surfaces (per quartile) following Rendu et al., 2014.**

The material is highly fragmented and no bone was found complete but the short bones. For instance, 90% of the long bone shaft fragments (%NRshaft US 02: 88.63%; US 04*upper*: 93.65%; US 04*lower*: 91.06; US 06: 89.78%; US 08: 92.43%) represents less than 25% of the total shaft length^9^. This fragmentation mostly occurred on green bone (77% of the bone exhibit green fractures) and conducts to a limited proportion of identified remains (36%) in the sample.

| **Ungulate size class** | **US02** | | **US04upper** | | **US04lower** | | **US06** | | **US08** | |
| --- | --- | --- | --- | --- | --- | --- | --- | --- | --- | --- |
|  | **NR** | **%NR** | **NR** | **%NR** | **NR** | **%NR** | **NR** | **%NR** | **NR** | **%NR** |
| **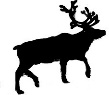** | 647 | 92% | 731 | 82% | 716 | 64% | 136 | 48% | 187 | 34% |
| **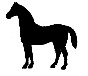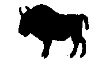** | 59 | 8% | 162 | 18% | 406 | 36% | 146 | 52% | 363 | 66% |

**SI2 Table4:** **Relative frequencies of medium and large size ungulates.**

|  | **US02** | **US04upper** | **US04lower** | **US06** | **US08** |
| --- | --- | --- | --- | --- | --- |
|  | NR | NR | NR | NR | NR |
| **Lagomorph** |  | 2 | 2 |  |  |
| **Canide** |  | 2 | 5 | 1 | 4 |
| **Hyena** |  | 3 | 7 | 9 | 4 |
| **Coprolite** |  | 1 | 1 |  | 1 |
| **Medium size carnivore** | 1 |  |  | 1 |  |
| **Small size carnivore** |  | 1 |  |  |  |
| **Large size carnivore** |  |  |  | 1 | 1 |
| **Equid** | 6 | 61 | 142 | 36 | 35 |
| **Mammouth** |  | 4 | 1 | 6 | 4 |
| **Capra** |  | 1 |  | 2 |  |
| **Chamois** | 1 |  |  |  |  |
| **Bovine** | 7 | 20 | 45 | 62 | 115 |
| **Wooly rhinoceros** | 4 |  | 1 | 3 | 1 |
| **Wild boar** |  |  | 1 | 1 |  |
| **Roe deer** |  |  | 1 |  |  |
| **Reindeer** | **438** | **469** | **440** | **118** | **131** |
| **Red deer** |  |  |  | 1 | 3 |
| **Megaceros** |  |  |  |  | 1 |

**SI2 Table5 : Faunal spectra par stratigraphic unit, including the results from** ^10^**. To allow the comparison, Vulpine and wolf have been reunited in the canid group; horse and hydruntinus are reunited in the Equids one. Only Identified remains are considered.**

|  | | **US02** | **US04upper** | **US04lower** | **US06** | **US08** |
| --- | --- | --- | --- | --- | --- | --- |
|  | **NRa** | **768** | **970** | **1164** | **407** | **673** |
| **NR Bone with carnivore marks** | **NR** | **4** | **21** | **52** | **42** | **80** |
|  | **%NR** | **0,52%** | **2,16%** | **4,47%** | **10,32%** | **11,89%** |
| **Digested bones** | NR | 1 | 11 | 39 | 35 | 63 |
|  | %NR | **0,13%** | **1,13%** | **3,35%** | **8,60%** | **9,36%** |
| ***Pit*** | NR | 2 | 7 | 13 | 6 | 9 |
|  | %NR | **0,26%** | **0,72%** | **1,12%** | **1,47%** | **1,34%** |
| ***Puncture*** | NR | - | 2 | 1 | - | 1 |
|  | %NR |  | **0,21%** | **0,09%** |  | **0,15%** |
| ***Groove*** | NR | 1 | - | 2 | 1 | 7 |
|  | %NR | **0,13%** |  | **0,17%** | **0,25%** | **1,04%** |
| **Gnawing bone** | NR | - | 1 | 3 | 2 | 10 |
|  | %NR |  | **0,10%** | **0,26%** | **0,49%** | **1,49%** |
| **Notches** | NR | - | 2 | 2 | 1 | 2 |
|  | %NR |  | **0,21%** | **0,17%** | **0,25%** | **0,30%** |

**SI2 Table6: Bones with carnivore modifications per layer.**

| **Taxa** | **US02** | **US04upper** | **US04lower** | **US06** | **US08** |
| --- | --- | --- | --- | --- | --- |
| **Fox** |  |  | 1 |  |  |
| **Hyena** |  | 1 | 3 | 1 |  |
| **Horse** |  | 6 | 10 | 3 | 4 |
| **Bovin** |  |  | 2 | 4 | 10 |
| **Reindeer** | 3 | 10 | 7 | 5 | 5 |
| **Megaceros** |  |  |  |  | 1 |
| **Mammoth** |  |  |  |  | 1 |
| **Medium size ungulate** |  |  |  | 2 | 4 |
| **Large size ungulate** | 1 |  | 10 | 2 | 18 |
| **Very large size ungulate** |  |  |  |  | 2 |
| **Mammal ind.** |  | 4 | 19 | 25 | 35 |

**SI2 Table7: Bones with carnivore modifications per taxon and per layer in NISP.**


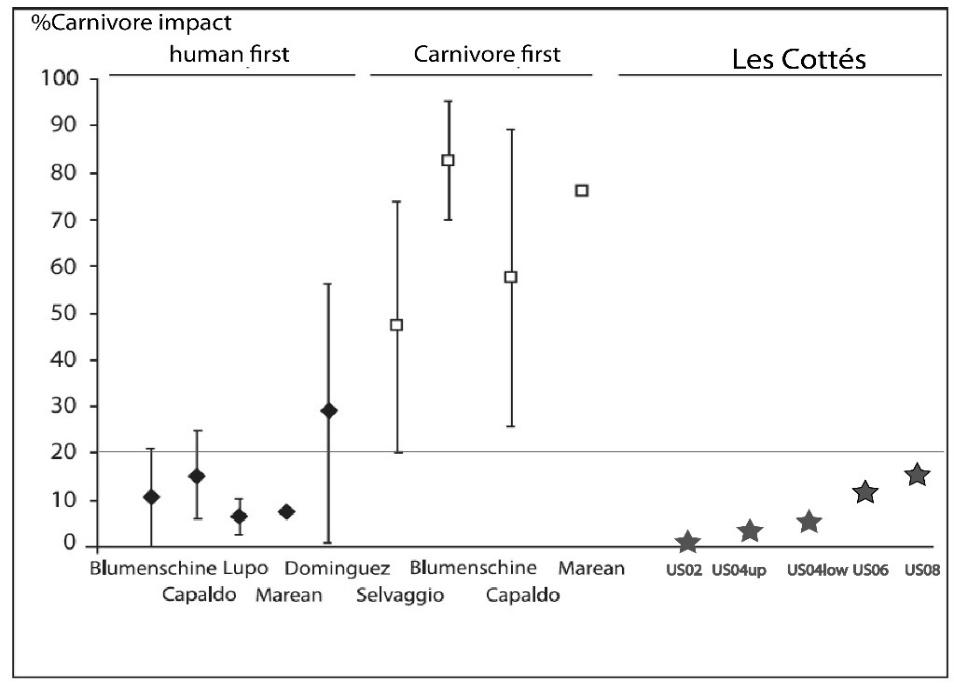


SI2 Figure 1: Comparison of the carnivore impact on the les Cottés assemblages with different experimental collections (modified after^11^) with man or carnivore acceding first to the carcasses^12–17^.

**SI2 Table8**: Anthropic modifications per taxa per layer. Expressed in NR.

|  | **US02** | **US04up** | **US04low** | **US06** | **US08** |
| --- | --- | --- | --- | --- | --- |
| **Lagomorphe** |  | 1 |  |  |  |
| **Vulpin** |  | 1 |  |  |  |
| **Large Carnivore** | 1 |  |  | 1 |  |
| **Hyena** |  |  |  | 1 |  |
| **Horse** |  | 10 | 10 | 1 | 3 |
| **Chamois** | 1 |  |  |  |  |
| **Bovin** | 3 | 4 | 12 | 2 | 21 |
| **Reindeer** | 82 | 107 | 110 | 14 | 26 |
| **Megaceros** |  |  |  |  | 1 |
| **Small ungulate** | 3 | 2 | 1 | 1 |  |
| **Med. ungulate** | 39 | 52 | 54 | 12 | 13 |
| **Large ungulate** | 4 | 23 | 80 | 15 | 29 |
| **Mammal** | 32 | 45 | 125 | 21 | 19 |

## Supplementary information 3 :

|  |  | **US02** | | | | **US04 upper** | | | | **US04 lower** | | | | **US06** | | | | **US08** | | | |
| --- | --- | --- | --- | --- | --- | --- | --- | --- | --- | --- | --- | --- | --- | --- | --- | --- | --- | --- | --- | --- | --- |
|  | **QSP** | **NISP** | **NME** | **MAU** | **%MAU** | **NISP** | **NME** | **MAU** | **%MAU** |  | **NME** | **MAU** | **%MAU** | **NISP** | **NME** | **MAU** |  | **NISP** | **NME** | **MAU** | **%MAU** |
| **Antler** | 2 | 49 | 4 | 2 |  | 43 | 2 | 1 | 29% | 10 | 3 | 1.50 | 25% | 4 |  | 0 | 0% | 0 |  | 0 | 0% |
| **CRANE** | 1 | 1 | 4 | 4 | 53% | 5 | 2 | 2 | 57% | 2 | 3 | 3.00 | 50% | 1 | 2 | 2 | 80% | 0 |  | 0 | 0% |
| **Mandible** | 2 | 8 | 3 | 1.5 | 20% | 19 | 2 | 1 | 29% | 16 | 2 | 1.00 | 17% | 2 | 1 | 0.5 | 20% | 4 | 3 | 1.5 | 60% |
| **ATLAS** | 1 |  | 0 | 0 | 0% |  |  | 0 | 0% |  |  | 0.00 | 0% | 0 |  | 0 | 0% | 0 | 0 | 0 | 0% |
| **AXIS** | 1 |  | 1 | 1 | 0% |  |  |  | 0% |  |  | 0.00 | 0% | 0 |  | 0 | 0% | 0 | 0 | 0 | 0% |
| **Cervical** | 6 |  | 0 | 0 | 0% | 2 | 2 | 0.333 | 10% | 1 | 1 | 0.17 | 3% | 0 |  | 0 | 0% | 2 | 2 | 0.333 | 13% |
| **Thoracic** | 13 | 1 | 1 | 0.077 | 1% | 4 | 3 | 0.231 | 7% | 6 | 1 | 0.08 | 1% | 1 | 1 | 0.077 | 3% | 8 | 6 | 0.462 | 18% |
| **Lumbar** | 6 |  | 0 | 0 | 0% | 2 | 1 | 0.167 | 5% | 3 | 1 | 0.17 | 3% | 1 | 1 | 0.167 | 7% | 1 | 1 | 0.167 | 7% |
| **Rib** | 26 | 21 | 7 | 0.269 | 4% | 23 | 3 | 0.115 | 3% | 31 | 3 | 0.12 | 2% | 4 | 3 | 0.115 | 5% | 8 | 2 | 0.077 | 3% |
| **Sternebre** | 10 |  | 0 | 0 | 0% |  | 0 | 0 | 0% |  | 0 | 0.00 | 0% | 0 | 0 | 0 | 0% | 0 |  | 0 | 0% |
| **Scapula** | 2 | 2 | 1 | 0.5 | 7% | 3 | 1 | 0.5 | 14% | 3 | 2 | 1.00 | 17% | 0 |  | 0 | 0% | 1 | 1 | 0.5 | 20% |
| **Humerus** | 2 | 29 | 9 | 4.5 | 60% | 22 | 7 | 3.5 | 100% | 19 | 5 | 2.50 | 42% | 6 | 4 | 2 | 80% | 8 | 4 | 2 | 80% |
| **Radio Ulna** | 2 | 29 | 5 | 2.5 | 33% | 49 | 6 | 3 | 86% | 49 | 6 | 3.00 | 50% | 3 | 2 | 1 | 40% | 13 | 4 | 2 | 80% |
| **Carpal** | 12 | 1 | 1 | 0.083 | 1% | 4 | 3 | 0.25 | 7% | 2 | 1 | 0.08 | 1% | 1 |  | 0 | 0% |  |  | 0 | 0% |
| **Metacarpal** | 2 | 16 | 4 | 2 | 27% | 29 | 4 | 2 | 57% | 34 | 3 | 1.50 | 25% | 3 | 2 | 1 | 40% | 4 | 2 | 1 | 40% |
| **Pelvis** | 2 |  | 0 | 0 | 0% | 4 | 2 | 1 | 29% | 2 | 1 | 0.50 | 8% | 1 | 1 | 0.5 | 20% | 1 | 1 | 0.5 | 20% |
| **Femur** | 2 | 31 | 7 | 3.5 | 47% | 25 | 6 | 3 | 86% | 21 | 2 | 1.00 | 17% | 8 | 5 | 2.5 | 100% | 8 | 4 | 2 | 80% |
| **Tibia** | 2 | 82 | 15 | 7.5 | 100% | 67 | 6 | 3 | 86% | 76 | 12 | 6.00 | 100% | 10 | 5 | 2.5 | 100% | 14 | 5 | 2.5 | 100% |
| **Patella** | 2 |  |  |  |  | 1 |  |  |  | 1 |  |  |  | 0 | 0 | 0 | 0 |  |  | 0 | 0% |
| **Tarsal** | 6 | 6 | 4 | 0.667 | 9% | 3 | 3 | 0.5 | 14% | 5 | 3 | 0.50 | 8% | 0 | 0 | 0 | 0% | 1 | 1 | 0.167 | 7% |
| **Maleolar bone** | 2 |  |  |  |  |  |  |  |  | 1 |  |  |  | 1 | 1 | 0.5 |  | 1 | 1 | 0.5 | 20% |
| **Metatarsal** | 2 | 82 | 7 | 3.5 | 47% | 64 | 3 | 1.5 | 43% | 66 | 5 | 2.50 | 42% | 10 | 2 | 1 | 40% | 15 | 5 | 2.5 | 100% |
| **Palanx 1** | 8 | 8 | 6 | 0.75 | 10% | 4 | 6 | 0.75 | 21% | 6 | 2 | 0.25 | 4% |  | 1 | 0.125 | 5% | 1 |  | 0 | 0% |
| **Palanx 2** | 8 | 2 | 2 | 0.25 | 3% | 4 | 3 | 0.375 | 11% | 1 | 1 | 0.13 | 2% | 11 | 0 | 0 | 0% |  |  | 0 | 0% |
| **Palanx 3** | 8 |  | 0 | 0 | 0% |  | 0 | 0 | 0% | 1 | 1 | 0.13 | 2% |  | 0 | 0 | 0% |  |  | 0 | 0% |
| **sesamoide** |  | 1 |  |  |  | 1 |  |  |  | 2 |  |  |  |  | 0 |  |  | 1 |  |  |  |
| **Phalanx access** | | 3 |  |  |  | 2 |  |  |  | 2 |  |  |  |  | 1 |  |  |  |  |  |  |
| **Metapodial ind.** | | 15 |  |  |  | 9 |  |  |  | 20 |  |  |  | 6 | 0 |  |  |  |  |  |  |

**SI3 table1: NME, MAU and %MAU per layers for the reindeer.**

|  |
| --- |
|  |
|  |
|  |
|  |

**SI3 figure1 : Differential preservation test: Reindeer relative skeletal representation (%MAU) compared to the bone density US 02 ddl : 46 ; rs=0.466, p<0.001 ; US 04upper ddl : 46 ; rs=0.365, p<0.01 ; US 04lower ddl : 46 ; rs=0.400, p<0.001 ; US 06 ddl : 46 ; rs=0.384, p<0.001; US 08 ddl : 46 ; rs=0.361, p<0.001.**

|  |  |
| --- | --- |
|  |  |
|  |  |
|  |  |
|  |  |

**SI3 figure2: Reindeer relative skeletal representation (%MAU) compared to the Oleic Acid value**^18^ **(a-e) and the Marrow Utility Index**^19^ **(f-j).** MUI US 02: 0.90206, p<0.001; US 04upper: 0.7838, p<0.001; US 04lower: 0.8612, p<0.001; US 06: 0.7744, p<0.001; US 08: 0.51, p<0.05; Oleic Acid: US 02: 0.9207, p<0.001; US 04upper: 0.8245, p<0.001; US 04lower: 0.8181, p<0.001; US 06: 0.9014, p<0.001; US 08: 0.9304, p<0.001.

**SI References :**

1. Rochebrune, R. *Les Troglodytes de La Gartempe. Fouilles de la grotte des Cottés*. (Imprimerie Charles Caurit, 1881).

2. Pradel, L. La grotte des Cottés commune de Saint-Pierre-de-Maillé (Vienne). *l’Anthropologie* **65,** 229–254 (1961).

3. Soressi, M. *et al.* *Les Cottés (Vienne). Nouveaux travaux sur l’un des gisements de référence pour la transition Paléolithique moyen/supérieur*. (Association des Publications Chauvinoises, 2010).

4. Talamo, S., Soressi, M., Roussel, M., Richards, M. & Hublin, J.-J. A radiocarbon chronology for the complete Middle to Upper Palaeolithic transitional sequence of Les Cottés (France). *Journal of Archaeological Science* **39,** 175–183 (2012).

5. Jacobs, Z., Li, B., Jankowski, N. & Soressi, M. Testing of a single grain OSL chronology across the Middle to Upper Palaeolithic transition at Les Cottés (France). *J. Archaeolog. Sci.* **54,** 110–122 (2015).

6. Hajdinjak, M. *et al.* Reconstructing the genetic history of late Neanderthals. *Nature* **555,** 652 (2018).

7. Rigaud, S. *et al.* Les pratiques ornementales à l’Aurignacien ancien dans le Centre-Ouest de la France: L’apport des fouilles récentes aux Cottés (Vienne). *Bulletin de la Société préhistorique française* **111,** 19–38 (2014).

8. White, R. *et al.* Technologies for the Control of Heat and Light in the Vézère Valley Aurignacian. *Current Anthropology* **58,** S288–S302 (2017).

9. Villa, P. & Mahieu, E. Breakage patterns of human long bones. *Journal of Human Evolution* **21,** 27–48 (1991).

10. Welker, F., Soressi, M., Rendu, W., Hublin, J.-J. & Collins, M. Using ZooMS to identify fragmentary bone from the Late Middle/Early Upper Palaeolithic sequence of Les Cottıfmmode\acutee\elseé\f is, France. *J. Archaeolog. Sci.* **54,** 279–286 (2015).

11. Costamagno, S. *et al.* Homme ou carnivores ? Protocole d’étude d’ensembles osseux mixtes : *Palethnologie* **1,** 372–400 (2009).

12. Lupo, K. D. & O’Connell, J. F. Cut and Tooth Mark Distributions on Large Animal Bones: Ethnoarchaeological Data from the Hadza and Their Implications For Current Ideas About Early Human Carnivory. *J. Archaeolog. Sci.* **29,** 85–109 (2002).

13. Selvaggio, M. M. Carnivore tooth marks and stone tool butchery marks on scavenged bones: archaeological implications. *J. Hum. Evol.* **27,** 215–228 (1994).

14. Marean, C. W. The Middle Stone Age at Die Kelders Cave 1, South Africa, , t. 38, fasc. 1, p. 3-5. *Journal of Human Evolution* **38,** 3–5 (2000).

15. Capaldo, S. D. Simulating the Formation of Dual-Patterned Archaeofaunal Assemblages with Experimental Control Samples. *J. Archaeolog. Sci.* **25,** 311–330 (1998).

16. Blumenschine, R. J. Percussion marks, tooth marks, and experimental determinations of the timing of hominid and carnivore access to long bones at FLK Zinjanthropus, Olduvai Gorge, Tanzania. *J. Hum. Evol.* **29,** 21–51 (1995).

17. Domingez-Rodrigo, M. Flesh availability and bone modifications in carcasses consumed by lions: palaeoecological relevance in hominid foraging patterns. *Palaeogeography, Palaeoclimatology, Palaeoecology* **149,** 373–388 (1999).

18. Morin, E. Fat composition and Nunamiut decision-making: a new look at the marrow and bone grease indices. *Journal of Archaeological Science* **34,** 69–82 (2007).

19. Jones, K. T. & Metcalfe, D. Bare bones archaeology: Bone marrow indices and efficiency. *Journal of Archaeological Science* **15,** 415–423 (1988).
